# Elliptical Polarization of Localized States in an Anisotropic Single GaAs Quantum Ring

**DOI:** 10.3390/nano13010184

**Published:** 2022-12-31

**Authors:** Seongho Park, Minju Kim, Inhong Kim, Robert A. Taylor, Jindong Song, Kwangseuk Kyhm

**Affiliations:** 1 Department of Opto/Cogno-Mechatronics Engineering, Research Center for Dielectric Advanced Matter Physic (RCDAMP), Pusan National University, Busan 46241, Republic of Korea; 2 Smart Gym-Based Translational Research Center for Active Senior’s Healthcare, Pukyong National University, Busan 48516, Republic of Korea; 3 Department of Physics, University of Oxford, Oxford OX1 3PU, UK; 4 Korea Institute of Science and Technology (KIST), Seoul 02792, Republic of Korea

**Keywords:** quantum ring, localized states, polarization, Stokes parameter

## Abstract

Localized states in an anisotropic single GaAs quantum ring were investigated in terms of polarization dependence of micro-photoluminescence spectrum at 5K. Given four Stokes parameters measured with a pair of linear polarizers and waveplates, the elliptical polarization states of two different vertical confinement states (k=1 and k=2) were compared with phase, rotation, and ellipticity angles. While the polarized emission intensity of the k=2 states becomes enhanced along [1,1,0] compared to that along [1,$\bar{1}$,0], the polarization asymmetry of the k=1 states shows the opposite result. We conclude the polarization state is determined by the shape of the lateral wavefunctions. In the k=2 state, crescent-like wavefunctions are strongly localized, but the k=1 state consists of two crescent-like wavefunctions, which are connected weakly through quantum tunneling.

## 1. Introduction

Semiconductor quantum rings (QRs) grown by the droplet epitaxy technique have gained a lot of interest during the recent two decades [1,2,3]. Regarding the closed loop structure, QRs can be an alternative platform to investigate the Aharonov–Bohm (AB) effect [4,5,6,7,8]. Although mesoscopic ring structures were mainly used to observe the AB effect, the fabricated size is of a sub-micron scale. Therefore, it requires extremely low temperatures (mK). However, the decreased size of semiconductor QRs (∼50 nm) enables the observation of the AB effect up to tens of Kelvin.

Due to the quantized orbital angular momentum states in a QR, fine levels appear between the lateral confinement levels. Interestingly, it is known that the energetic arrangement order of the different angular momentum states changes for increasing an external magnetic field. Therefore, the lowest energy levels show oscillations for the external magnetic field, and this can be verified through magneto micro-photoluminescence (micro-PL) spectroscopy [7,8].

Nevertheless, it is also known that the structure of QRs is different from the ideal torus shape. According to the atomic force microscopy image, the height of QRs is anisotropic similar to a volcano-like structure. In this case, the wavefunction can be localized with a crescent-like shape [9,10,11,12,13], and two crescent-like localized states can be present in the same QR. If the two localized states are separated, it is known that the two crescent-like wavefunctions can be connected through quantum tunneling by applying external energy. However, it is difficult to know whether a singly connected loop of carriers is given in a QR in the absence of external energy. Additionally, the transmission electron microscopy image of QRs shows that the lateral shape of QRs is elliptical. Based on the Miller index, the elongated direction can be denoted by [1,$\bar{1}$,0] [14,15,16,17], which is perpendicular to [1,1,0]. Therefore, the loop shape of a QR is not of an isotropic circle even if the two localized states are connected.

When electrons and holes are considered separately, the separate adiabatic potential valley of electrons and holes can be obtained regarding the height and lateral shape of QRs, whereby the lateral wavefunctions for electrons and holes are also obtained separately. This simplified model is convenient for estimating the degree of localization. Because PL is generated by electron–hole pairs, the Coulomb interaction needs to be considered. From a practical point of view, the exact Coulomb interaction in anisotropic confinement structures requires massive calculation. However, in the case of strongly confined quantum dots, the confinement energy is still dominant compared to the Coulomb energy, although the effective Coulomb interaction is known to be enhanced. Therefore, the exact energy level of electron–hole pairs can be refined by adding the effective Coulomb term on the separate energy levels of independent electrons and holes, and a lateral anisotropy of the exciton dipole can be estimated by the wavefunction overlap between the separate wavefunctions of electrons and holes. For optical experiments, elliptical polarization analysis is a useful tool to investigate the lateral shape of the wavefunction. While a pair of linear polarizers can reveal lateral asymmetry, it is not enough to identify the exact polarization state. In this work, we studied the elliptical polarization of the micro-PL spectrum in a single QR by analyzing four Stokes parameters, and the polarization state was fully characterized in terms of phase, rotation, and ellipticity angles. The result was also consistent with the lateral shape of the localized states.

## 2. Materials and Methods

Self-assembled QR structures were grown using the droplet epitaxy method. Initially, we prepared the substrate, where 100 nm thick GaAs buffer layers and a 50 nm thick AlGaAs barrier layer were grown on a GaAs substrate. For a metal-stabilized surface environment, any surface oxides on the substrate were thermally removed. Afterward, 3 ML Ga metallic atoms were deposited on the substrate to form the droplets. The sequential addition of As tetramer for 30 s and 2 min leads to the spontaneous formation of nanometer-scale GaAs QR and crystallization, respectively. Finally, GaAs QR structures were capped with a 50 nm thick AlGaAs barrier layer and annealed at 600 °C to enhance the optical properties.

Briefly, 532 nm Nd:YAG picosecond pulsed laser (∼6 ps) operating at 50 MHz repetition rate was used for excitation. For polarization analysis, a pair of a half-waveplate and a quarter-waveplate were used. The sample was cooled down to 5 K in a helium flow cryostat, and the time-integrated PL spectrum was measured with a charge-coupled device (CCD) camera using a monochromator. Because a slit can perform as an efficient linear polarizer, the polarized light perpendicular to the slit direction was not completely removed. Hence, we put an additional linear polarizer in front of the slit entrance, which was parallel to the slit direction. A long-pass filter (>532 nm) was also used to remove the scattered laser lights.

## 3. Results and Discussion

**Figure 1:**
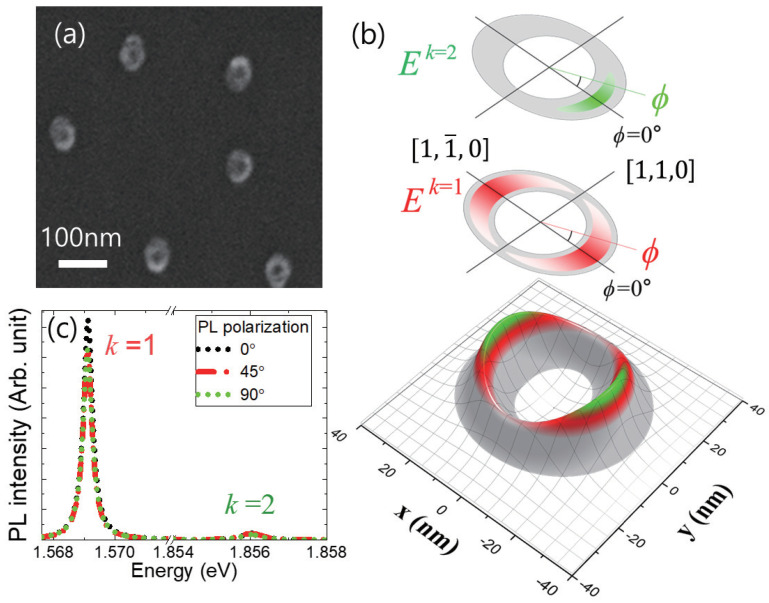
(**a**) FESEM image of uncapped GaAs QRs shows the density of QRs is enough low to perform micro-PL. (**b**) The structure of an anisotropic QR is shown theoretically regarding the AFM, where the lateral plane is defined in xy plane, and the vertical direction (*z*-axis) corresponds to the height of the QR. Two different crescent-like wavefuntions of vertically localized states (k=1 and k=2) are also shown schematically. (**c**) Two miicro-PL spectra were obtained through three linear analyzer angles (0°, 45°, and 90°), which correspond to the k=1 states and k=2 states.

In Figure 1a, an image of uncapped QRs was obtained by a field emission scanning electron microscope (FESEM). We found the density of QRs (∼4 × 10${}^{9}$ cm${}^{-2}$) was low enough to measure the emission spectrum from a single QR. As shown schematically in Figure 1b, it was known that the lateral shape of QRs is elliptical, which is elongated along [1,$\bar{1}$,0], and the height is anisotropic similar to a volcano-like shape. As a result, the excitons in a QR are likely localized.

The confinement energy levels of the excitons can be calculated through the adiabatic potential, where the height variation is considered with the adiabatic approximation. In this case, the slowly varying lateral wavefunction becomes separated from the fast-varying vertical wavefunction. Therefore, the adiabatic potential is given by the height variation, and the lateral wavefunction is determined by the adiabatic potential. Given the average height (6∼10 nm) of QRs, we found there are two vertical confinement states, which are denoted by the vertical quantum number of *k*.

For the excited vertical state of k=2, the exciton wavefunction is localized similarly to a crescent shape. Specifically, the azimuthal angle ϕ can be defined with respect to the symmetric axis of the crescent-like wavefunction (ϕ=0°). In Figure 1c, the PL spectrum from the k=2 state was observed near 1.856 eV, and we found that the PL intensity along the symmetric axis (ϕ=0°) was suppressed compared to that at the perpendicular angle (ϕ=90°). Regarding the rim width (∼20 nm) and the diameter (∼50 nm), the exciton oscillator strength is expected to increase with the increase in the confinement size [18,19]. Therefore, the oscillator strength for the ϕ = 90° orientation is large compared to that for the perpendicular direction (ϕ=0°).

On the other hand, the adiabatic potential structure of the k=1 state is known to be less stiff compared to that of the k=2 state. In this case, the extended two crescent-like wavefunctions can be overlapped through tunneling, thus giving rise to an elliptical donut shape. Therefore, The PL spectrum at 1.569 eV can be attributed to the k=1 state (Figure 1c), and the polarization dependence of the k=1 states becomes different from that of the k=2 states. However, this issue in a QR has never been investigated.

In Figure 1c, the PL spectra of the k=1 and k=2 states were measured with the two perpendicular analyzer angles (0° and 90°) in order to investigate the polarization anisotropy. It is noticeable that the orientation of the 0° analyzer angle is arbitrary, where the selection depends on the alignment condition of analyzers, and the additional measurement at 45° is a useful reference to estimate the angle difference (ψ) between the symmetric axis of a QR and the analyzer angle, which can be either positive or negative. Although a pair of perpendicular polarization angles are often used to study the polarization dependence of PL spectra, further analysis is necessary to find the major axis of an elliptically polarized state. In other words, the 0° angle of our analyzer does not guarantee the major axis of an elliptical quantum ring.

**Figure 2:**
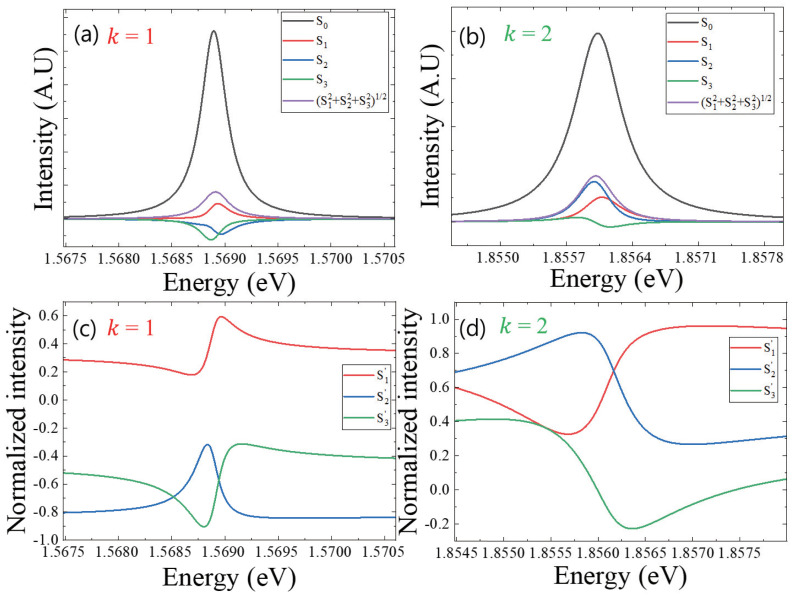
The spectrum of four Stokes parameters ($S_{0},S_{1},S_{2},S_{3}$) and magnitude ($S=\sqrt{S_{1}^{2}+S_{2}^{2}+S_{3}^{2}}$) were obtained separately in k=1 (**a**) and k=2 states (**b**). Regarding the degree of coherence polarization ($\frac{S}{S_{0}}$), three spectrum of normalized Stokes parameters ($S_{1}^{{}^{\prime }}=S_{1}\;$⁄S, $S_{2}^{{}^{\prime }}=S_{2}\;$⁄S, $S_{3}^{{}^{\prime }}=S_{3}\;$⁄S) were obtained for the k=1 (**c**) and k=2 states (**d**), respectively.

When a polarization state of monochromatic light with an angular frequency ω is described in a two-dimensional plane, the two perpendicular components of an electric field are given by

(1) $$E_{x}\left(t\right)=E_{0x}\cos\left(\omega t+\delta_{x}\right),$$

(2) $$E_{y}\left(t\right)=E_{0y}\cos\left(\omega t+\delta_{y}\right),$$

where the amplitude ($E_{0x},E_{0y}$) and the phase ($\delta_{x},\delta_{y}$) are defined for the *x*- and *y*- axis, respectively. It is noticeable that the two components of electric fields are measurable from the PL spectrum. Therefore, the Jones vector ($E_{x}\left(\hbar \omega \right),E_{y}\left(\hbar \omega \right)$) has spectral dependence, where both amplitude and phase depend on emission energy (ℏω). Those are associated with four Stokes parameters as [20]

(3) $$S_{0}=E_{0x}^{2}+E_{0y}^{2},$$

(4) $$S_{1}=E_{0x}^{2}-E_{0y}^{2},$$

(5) $$S_{2}=2E_{0x}E_{0y}\cos\delta ,$$

(6) $$S_{3}=2E_{0x}E_{0y}\sin\delta ,$$

where $\delta =\delta_{y}-\delta_{x}$. To obtain the Stokes parameters experimentally, we used a linear polarizer and waveplates. In the first step, the polarized PL passes through a quarter-waveplate to induce a retardation of the phase factor. In the second step, a half-waveplate is adjusted before the linear polarizer, which was optimized to the monochromator grating. Finally, regarding the measured angle and phase retardation, the four Stokes parameters can be obtained. In Figure 2a,b, the four Stokes parameter spectra are shown for the PL spectra of the k=1 and k=2 states, respectively. Because $S_{0}$ is the total PL intensity, the square sum of polarized components $S=\sqrt{S_{1}^{2}+S_{2}^{2}+S_{3}^{2}}$ is less than $S_{0}$. Therefore, the PL coherence can be considered in terms of the degree of coherence polarization (*DCP*),

(7) $$DCP=\frac{\sqrt{S_{1}^{2}+S_{2}^{2}+S_{3}^{2}}}{S_{0}}.$$

For k=1 and k=2 states, we obtained *DCP*∼0.15 and ∼0.2, respectively. The poor coherence may support the presence of fine energy levels in the QR system. Compared to two- and three-dimensional confinement structures, quantum dots show a relatively long coherence. This was attributed to the separate energy levels, where the scattering with acoustic phonons is suppressed for intra-relaxation. However, the level spacing between the levels in QR is fine with sub-meV, and this causes various inelastic scattering. Recently, it has been found that the coherence time of a single QRs is under 30 ps [21].

To obtain a relative ratio between the polarized components, the Stokes parameters were normalized, where the three Stokes components are divided by the total polarized intensity (*S*), i.e., $S_{1}^{{}^{\prime }}=S_{1}\;$⁄S, $S_{2}^{{}^{\prime }}=S_{2}\;$⁄S, $S_{3}^{{}^{\prime }}=S_{3}\;$⁄S. Positive/negative values of the three normalized Stokes parameters $S_{1}^{{}^{\prime }}$, $S_{2}^{{}^{\prime }}$, and $S_{3}^{{}^{\prime }}$ represent horizontal/vertical linear, diagonal/anti-diagonal linear, and right/left circular polarization, respectively. For the k=1 state in Figure 2c, $S_{2}^{{}^{\prime }}$ and $S_{3}^{{}^{\prime }}$ are all negative over the spectrum with a large magnitude compared to positive small $S_{1}^{{}^{\prime }}$. On the other hand, for the k=2 state in Figure 2d, $S_{1}^{{}^{\prime }}$ and $S_{2}^{{}^{\prime }}$ show positive with partially negative $S_{3}^{{}^{\prime }}$. These results are in agreement with the localized shape of k=2, where the shape anisotropy gives rise to a strong degree of linear polarization. Interestingly, it is noticeable that the value of $S_{3}^{{}^{\prime }}$ on k=2 state changes from positive to negative near 1.856 eV. This result implies that the PL polarization changes from right circular to left circular polarization.

**Figure 3:**
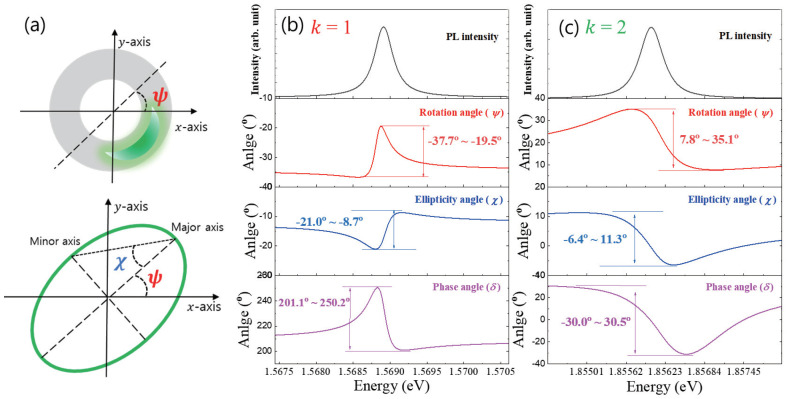
(**a**) A crescent−like localized wavefunction gives rise to an elliptical polarization. (**b**) Three spectra of polarization parameters (ψ, χ, and δ) were obtained from the PL spectrum of the k=1 state. Likewise, three spectra of polarization parameters in the k=2 state were plotted (**c**).

As shown schematically In Figure 3a, an elliptical polarization is emitted from a crescent-like localized state in the anisotropic QR structure, and the geometrical feature is analyzed with two perpendicular axes of *x* and *y*. When a phase difference $\delta =\delta_{y}-\delta_{x}$ is involved between the component phases ($\delta_{x}$ and $\delta_{y}$) of perpendicular electric fields ($E_{x}$ and $E_{y}$), the polarization becomes elliptical. Given the two perpendicular axes of *x* and *y*, the geometric shape can be represented by the two parameters of rotation angle (ψ) and ellipticity angle (χ). Although the orientation of the *x*-axis is arbitrary, the two symmetric axes of elliptical polarization are associated with the shape of crescent-like localized states. To present the symmetry of an ellipse, two perpendicular orientations of major and minor axes are defined. Regarding the size dependence of exciton oscillator strength, the radial direction corresponds to the minor axis, and PL intensity becomes dominant along the direction perpendicular to the minor axis. Therefore, the rotation angle (ψ) is an angle between the major axis and the *x*-axis, and the ellipticity angle (χ) is also determined by the major and minor axes. Given the three measured parameters of $S{{}^{{}^{\prime }}}_{1}$, $S{{}^{{}^{\prime }}}_{2}$, and $S{{}^{{}^{\prime }}}_{3}$, we obtained three polarization parameters (ψ, χ, and δ) using

(8) $$\psi =\frac{1}{2}\tan^{-1}\left(\frac{S_{2}^{{}^{\prime }}}{S_{1}^{{}^{\prime }}}\right),$$

(9) $$\chi =\frac{1}{2}\sin^{-1}(S_{3}^{{}^{\prime }}),$$

(10) $$\delta =\frac{1}{2}\tan^{-1}\left(\frac{S_{3}^{{}^{\prime }}}{S_{2}^{{}^{\prime }}}\right).$$

As shown in Figure 2, we obtained the spectrum of normalized Stokes parameters (${S^{{}^{\prime }}}_{1}$, $S{{}^{{}^{\prime }}}_{2}$, and $S{{}^{{}^{\prime }}}_{3}$). Therefore, the polarization parameters (ψ, χ, and δ) also depend on spectral energy. For k=1 (Figure 3b) and k=2 (Figure 3c), the spectra of three polarization parameters were obtained from the spectrum of normalized Stokes parameters (${S^{{}^{\prime }}}_{1}$, $S{{}^{{}^{\prime }}}_{2}$, and $S{{}^{{}^{\prime }}}_{3}$), respectively. In the rotation angle (ψ) spectrum, the k=1 state shows negative values, but the k=2 state shows positive values. Regarding Equation (8), this result originates from the sign of $S{{}^{{}^{\prime }}}_{1}$ and $S{{}^{{}^{\prime }}}_{2}$. In the case of the k=2 state, both the spectra of $S{{}^{{}^{\prime }}}_{1}$ and $S{{}^{{}^{\prime }}}_{2}$ show positive, but the signs of $S{{}^{{}^{\prime }}}_{1}$ and $S{{}^{{}^{\prime }}}_{2}$ spectra for the k=1 state are different. Likewise, the sign of δ is governed by the two parameters of $S{{}^{{}^{\prime }}}_{2}$ and $S{{}^{{}^{\prime }}}_{3}$, as shown in Equation (10). On the other hand, the sign of the ellipticity angle (χ) is directly associated with $S{{}^{{}^{\prime }}}_{3}$ according to Equation (9). For a selected emission wavelength, an elliptical polarization geometry can be visualized in x−y coordinates. Therefore, the spectrum of polarization parameters enables a comparison of the elliptical shape for different emission wavelengths.

**Figure 4:**
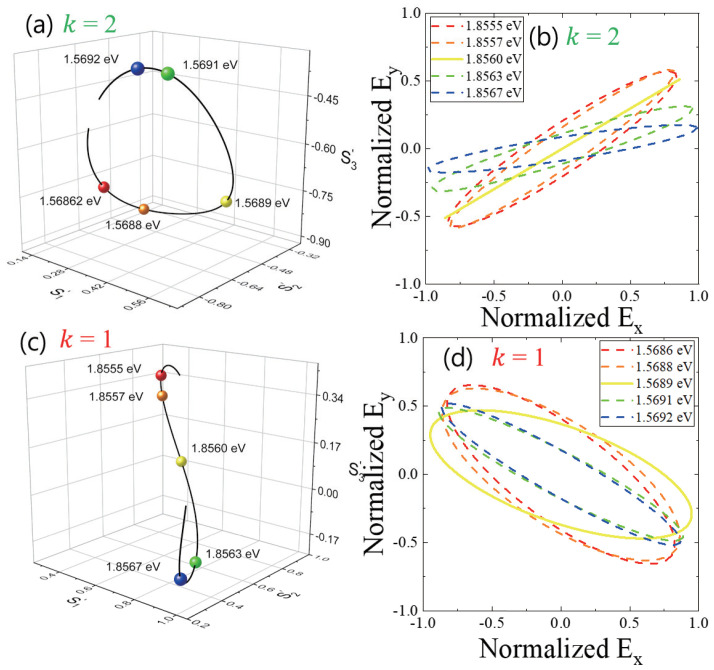
Various polarization states selected near the PL spectrum of the k=2 state are plotted in Poincaré space (**a**), and the corresponding lateral elliptical polarization shapes are also shown in *x*-*y* coordinates (**b**). Normalized Stokes parameters according to different spectrum energy values in k=2 state and k=1 state are mapped on a Poincaré sphere, respectively. (**c**,**d**) For the k=1 states, various elliptical polarization states selected at different spectrum energy values are also plotted in Poincaré space and *x*-*y* coordinates.

In Figure 4a, the polarization states near the PL spectrum of the k=2 state are plotted in Poincaré space, where the coordinates consist of the three normalized Stokes parameters (${S^{{}^{\prime }}}_{1}$, $S{{}^{{}^{\prime }}}_{2}$, and $S{{}^{{}^{\prime }}}_{3}$), and ${S^{{}^{\prime }}}_{1}$, $S{{}^{{}^{\prime }}}_{2}$, and $S{{}^{{}^{\prime }}}_{3}$ represent horizontal/vertical ($\hat{x}$, $\hat{y}$), diagonal/anti-diagonal ($\frac{1}{\sqrt{2}}\hat{x}\pm \frac{1}{\sqrt{2}}\hat{y}$), and right-circular/left-circular ($\frac{1}{\sqrt{2}}\hat{x}\pm i\frac{1}{\sqrt{2}}\hat{y}$) polarization state, respectively. Because we normalized the three parameters (${S^{{}^{\prime }2}}_{1}+S{{}^{{}^{\prime }2}}_{2}+S{{}^{{}^{\prime }2}}_{3}=1$), all of the polarization states are on the surface of a unit sphere. Therefore, different polarization states can be visualized on the surface. Given the five selected energy near the PL spectrum (1.8555 eV, 1.8557 eV, 1.8560 eV, 1.8563 eV, and 1.8567 eV), the different polarization states are mapped in three-dimensional Poincaré space. In Figure 4b, the various polarization shapes of the k=2 states are shown in *x*-*y* coordinates, which are selected from the PL spectrum. The rotation angle (ψ = 7.8°∼35.1°) and the ellipticity angle (χ = −6.4°∼11.3°) of the five emission energy states vary. The elliptical polarization shape is associated with the crescent-like shape of the k=2 localized state. Provided that the GaAs quantum dot size is larger than 5 nm, it is known that the exciton oscillator strength (*f*) increases with the confinement size (*L*), where the specific power factor is approximated to be 1.33 (*f* ∼ $L^{1.33}$) [22]. According to the theoretical work, the waist size of the crescent-like k=2 localized state is known to be 20 nm, which corresponds to the direction of ϕ = 90° in Figure 1b. Therefore, the exciton oscillator strength along ϕ = 90° is large compared to that along ϕ = 0°. Consequently, the emission intensity along ϕ = 90° becomes enhanced compared to that along ϕ = 0°. Likewise, the polarization states near the PL spectrum of the k=1 state are plotted in the Poincaré space, as shown in Figure 4c. We also selected five emission energy values near the k=1 PL spectrum (1.5682 eV, 1.5689 eV, 1.5688 eV, 1.5691 eV, and 1.5692 eV), and the polarization shapes are shown in the x−y coordinates in Figure 4d. Interestingly, all show negative rotation angles (ψ = −37.7°∼−19.5°), and the ellipticity angles (χ = −21.0°∼−8.7°) are enhanced compared to those of the k=2 state. This novel polarization feature is associated with the wavefunction shape of the k=1 state. In the case of the k=2 state, the wavefunction is strongly localized. Therefore, two crescent-like wavefunctions are present in an the anisotropic QR, but they are separated spatially. On the other hand, the two localized states of the k=1 states can be connected through quantum tunneling because the adiabatic potential valley of the k=1 is relatively less stiff than that of the k=2. Due to the connected region, the wavefunction area is also increased. Additionally, the lateral shape of QRs is known to be elongated along ϕ = 0° in Figure 1b. Consequently, the oscillator strength along ϕ = 0° becomes significantly enhanced and larger than the oscillator strength along ϕ = 90°. This can also explain the negative rotation angles of the k=1 state. It is noticeable that the wavefunction shape also depends on the confinement energy. Therefore, the oscillator strength along ϕ = 90° also changes for different emission energy values.

## 4. Conclusions

Given the two different localized states (k=1 and k=2) of QRs, which originate from the vertical confinement states, we measured the micro-PL spectrum from a single QR, and the elliptical polarization was fully characterized using four Stokes parameters. We found that the k=1 and k=2 states show a significantly different polarization shape in the xy coordinates, and this result can be attributed to the lateral shape of the localized wavefunctions. In the state with k=2, elliptical polarization analysis reveals that two crescent-like wavefunctions are localized separately in two halves of the ring. On the other hand, the lateral wavefunctions of the k=1 state consist of two weakly connected crescents as a result of quantum tunneling. Therefore, we conclude that two kinds of lateral wavefunction shapes coexist in the same QR, namely a localized crescent and a closed loop.

## Data Availability

The data presented in this study are available on request from the corresponding author.
